# A study of market segmentation, government competition, and public service efficiency in China: Based on a semi-parametric spatial lag model

**DOI:** 10.1371/journal.pone.0297446

**Published:** 2024-04-16

**Authors:** Lan Yao, Ruoyu Luo, Xiaoqin Yi

**Affiliations:** 1 School of Public Administration, University of Electronic Science and Technology of China, Chengdu, Sichuan, China; 2 Sichuan Institute of Industry and Information Technology, Chengdu, Sichuan Province, China; Universiti Malaysia Sabah, MALAYSIA

## Abstract

Despite significant growth in fiscal expenditure, the overall level of public services in China remains inadequate. One approach to improving government public service efficiency from the perspective of management psychology is to strengthen government competition. However, only a few studies have explored the improvement of public service efficiency through government competition, with even fewer addressing the phenomena of market segmentation and spatial effects that accompany the process of government competition. This paper aims to fill this research gap by examining the effects of government competition and market segmentation on public service efficiency, as well as their spatial disparities. We initially employs the DEA method to assess the efficiency of public services based on inputs and outputs, and examines its spatial variations. Subsequently, a semi-parametric spatial lag panel model is utilized to validate the effects of market segmentation and government competition on public service efficiency. Our findings indicate that inter-provincial market segmentation leads to a decline in public service efficiency. Moreover, the influence of horizontal competition between local governments on public service efficiency varies depending on the degree of positive and negative effects in their competition dynamics. The impact of vertical competition between central and local governments on public service efficiency is influenced by the degree of fiscal decentralization. When the level of fiscal decentralization is below 0.808, vertical competition between central and local governments has a promoting effect on public service efficiency. However, when the degree of fiscal decentralization exceeds 0.08, this promoting effect weakens and gradually transforms into a negative influence. The insights and evidence provided by this study offer valuable guidance for for effectively reshaping the fiscal relations between the central and local governments in China and improving public service efficiency in the context of a new round of fiscal and tax system reforms.

## 1 Introduction

**Fig 1:**
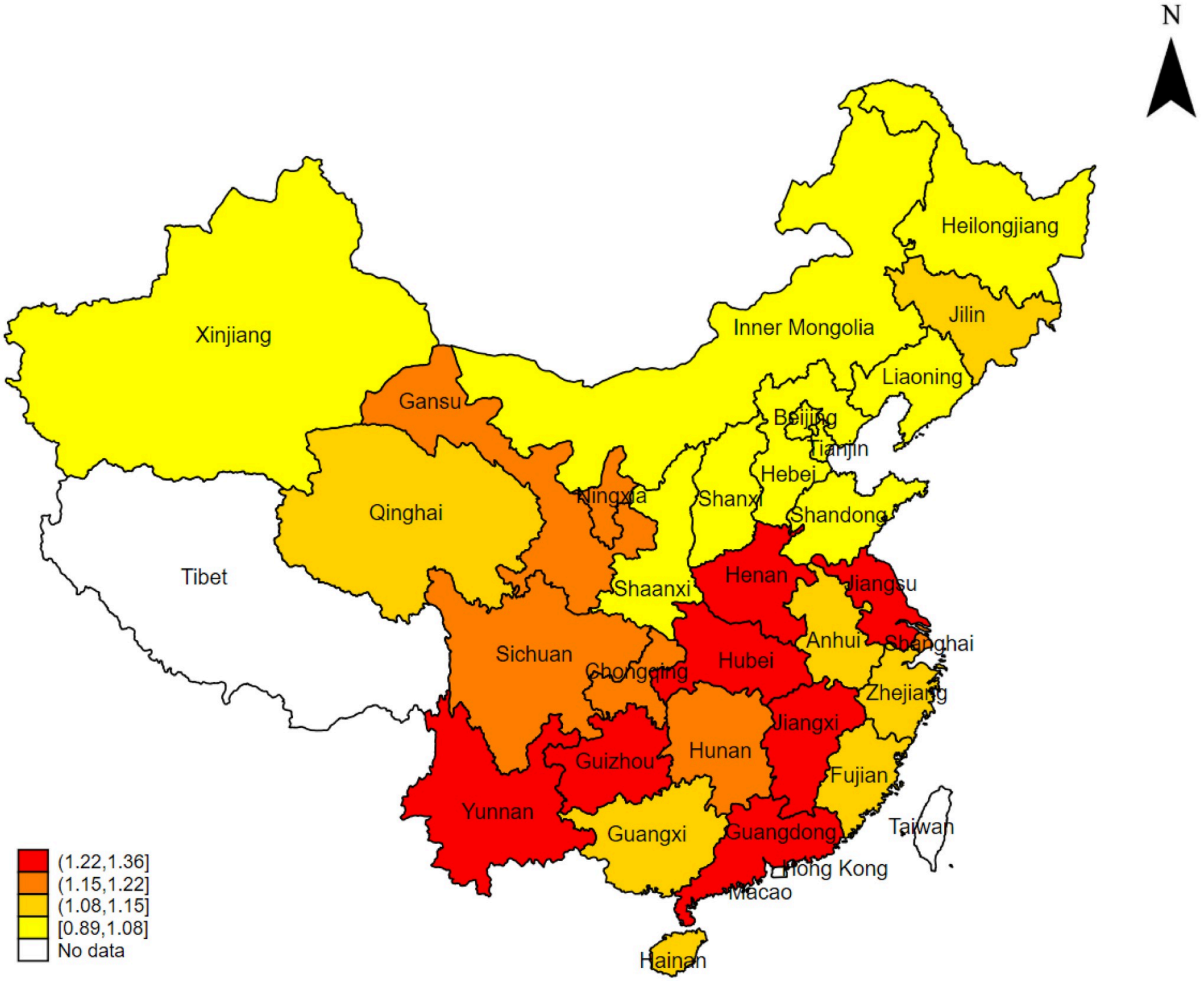


Over the past 40 years of reform and opening up, China’s Gross Domestic Product (GDP) has skyrocketed from 367.87 billion yuan in 1978 to 121.02 trillion yuan in 2022. Concurrently, the proportion of government fiscal expenditure to GDP has increased from 12% in 1994 to 22% in 2022. However, in sharp contrast to these figures, the public services provided by the government that are related to people’s well-being, such as education, healthcare, and social security, have increasingly failed to meet the growing demands of the population [1]. The “2022 Survey on the Development Index of Modern Public Services in China” quantitatively evaluated public satisfaction with various aspects of public services, based on the subjective perceptions of the general public. The results revealed that the current level of public services in China is not satisfactory, with an overall score of 65, indicating a decline compared to the score of 66.5 in 2009 [2]. Moreover, there are several challenges in the field of public services in China, including a shortage of high-quality resources, inadequate cooperation mechanisms among supply entities, and regional disparities in service capacity. These factors contribute to low efficiency and an overall lower level of public service provision in the country. As shown in Fig 1, which presents the public service efficiency levels of 30 Chinese provinces in 2009, it can be observed that the southeastern provinces exhibit higher public service efficiency compared to those in the northwestern region, highlighting significant regional imbalances in public service provision in China. Why is the overall level of public service in China still inadequate despite the high total and incremental government fiscal expenditure? Are these phenomena related to the efficiency of local government fiscal expenditure in China? Answering these questions requires a discussion on China’s fiscal system. The fiscal management system in China has undergone a transformation from centralization to decentralization, and from administrative decentralization to fiscal decentralization. Under the fiscal decentralization system, local governments have obtained autonomy granted by the central government. With the free flow of local resources and factors of production, as well as the premise of residents “voting with their feet,” fiscal decentralization can trigger regional competition among local governments. Is the competition among local governments resulting from fiscal decentralization beneficial for improving the level of public services? The first-generation theory of fiscal decentralization suggests that local governments, in their pursuit of public support, may allocate fiscal expenditures towards essential public services, thereby enhancing overall social welfare [3]. While scholars like Qian and Roland, representing the second generation of fiscal decentralization theory, argue that local governments, in their pursuit of fiscal transfers and resource accumulation, may overlook the welfare of the public, leading to a lower efficiency in public service provision [4]. However, in the specific context of China’s national conditions, how does the competition among local governments resulting from fiscal decentralization impact public service efficiency? Studying this issue holds significant theoretical and practical significance for promoting fiscal system reform in China, establishing a predominantly service-oriented public finance system, and improving public service efficiency.

Moreover, there is a noteworthy phenomenon where local governments, in order to gain victory in regional competition and secure economic and political benefits, may impose trade barriers or implement invisible preferential policies to protect their key industries, leading to the emergence of market segmentation in China [5]. Additionally, the uneven distribution of regional resources in China has led to significant disparities in economic development among provinces, further exacerbating market segmentation. Market segmentation severely affects the efficiency of resource allocation by local governments [6], including the allocation of public service resources. Against this backdrop, the following questions are raised in this study: How does government competition affect public service efficiency under the fiscal decentralization system? What impact does market segmentation caused by horizontal competition among governments have on the efficiency of public services? Are there spatial differences in the effects of government competition and market segmentation on public service efficiency? Addressing these questions serves as the motivation for this research.

## 2 Literature review

Although few scholars have explored the combination of government competition, market segmentation, and public service efficiency under fiscal decentralization, there is considerable research on the relationship between fiscal decentralization and public service efficiency, as well as on the relationship between local competition and market segmentation. These studies provide valuable insights for studying the impact of government competition and market segmentation on public service efficiency in this article. The relevant studies can be categorized as follows: research on public service efficiency; the relationship between local competition under fiscal decentralization and public service efficiency; the relationship between local competition, market segmentation, and public service efficiency.

### 2.1 Public service efficiency

The concept of public service efficiency can be traced back to welfare economics, with its core focus on the cost-benefit ratio, fairness, and their relationship. Samuelson defined and substantiated the notion of public service efficiency by stating that “the marginal rate of transformation of public to private goods equals the sum of the marginal rates of substitution.” [7] Academic research on public service efficiency has concentrated on three aspects: measurement of public service efficiency, factors influencing public service efficiency, and distinctive features of public service efficiency. Currently, the most commonly used method for measuring public service efficiency is Data Envelopment Analysis (DEA). As an optimization linear programming approach, it has been widely used in efficiency analysis in various public or private sectors [8]. Savas proposed measurement criteria for public service efficiency from four aspects: utilization, input, output, and demand. This method has been widely applied to measure specific areas of public services [9]. For example, Caroline and Campos applied DEA to evaluate the efficiency of public health services [10, 11]. Richard used DEA to evaluate the efficiency of public services in key areas such as public health and education in OECD countries, revealing significant differences in supply efficiency among countries [12]. Chen and Lai analyzed the public service efficiency of 31 provinces in China using a three-stage DEA model [13]. Yu found that the overall technical efficiency level of public services in China was only 0.760, indicating low efficiency [14]. Temporally, efficiency showed a “U”-shaped trend, while spatially, it exhibited a “step-like” pattern with higher efficiency in the east and lower efficiency in the west. In addition, some scholars have used Stochastic Frontier Analysis (SFA) to study public service efficiency. However, compared to SFA, DEA is better suited for studying public service efficiency as it effectively avoids the issues of model specification errors. Therefore, in this study, we utilize the DEA method based on an input-output perspective to measure the efficiency of public services across 30 provinces in China.

Scholars have differing opinions on the factors that influence public service efficiency. Buchanan studying the United States as a sample, found that the improvement of the supply efficiency of public goods relies on the increase in residents’ income levels [15]. However, Baumol believed that an increase in income levels would increase the supply cost of public services, leading to a decrease in supply efficiency [16]. Vanden argued that regions with higher levels of economic development tend to waste public resources [17]. Qin and Yang reached similar conclusions to Vanden’s, attributing it to increased supply costs of public services corresponding to the growth of local income levels [1]. Athanasso and Triantis found that population density has a negative impact on public service efficiency [18], while Grossman argued that there is a scale effect scale in government-provided public services, with decreasing costs of government management and supervision as the population increases [19]. Afonso, Xin and Chen, and others believe that government policies, economic development, industrial strategies, urbanization, and foreign direct investment are important factors driving the provision of basic public services [20, 21]. Some scholars also highlight factors such as promotion pressure, regional policies, institutional and technological innovation, and openness to international trade as influencing factors on the provision of basic public services [22–24].

Compared to Chinese scholars, international research pays less attention to regional differences in public service efficiency. Only a few studies have focused on the spatial distribution fairness of public services [9] and the strategic interactions among local governments, where the efficiency of public services in one region is influenced by the fiscal expenditures of neighboring regions [25]. Many Chinese scholars have explored the spatial convergence of public service efficiency through modeling. For example, convergence models are used to explore the convergence or divergence characteristics of basic public service levels over time [21, 26]. Research found significant *α*-convergence and *β*-convergence features in the basic public services among the three major regions in China [26]. Most studies have found that the efficiency of basic public services in China is trending upward but at a relatively low level, suggesting there is significant room for improvement. Moreover, there is a notable “higher in the east and lower in the west” regional disparity in public service efficiency.

### 2.2 Government competition and public service efficiency under fiscal decentralization

Scholars have conducted in-depth research from various perspectives on the relationship between fiscal decentralization and public service efficiency. However, due to differences in measurement methods, underlying assumptions, empirical models, and estimation techniques for fiscal decentralization and public service efficiency, empirical analysis has yielded two opposing views: local government competition under fiscal decentralization may either have a positive or negative correlation with public service efficiency. Regarding the potential positive impact of local government competition under fiscal decentralization on public service efficiency, several arguments can be highlighted: (1) fiscal decentralization improves the efficiency of public service provision through preference matching and optimal resource allocation. By fostering closer connections between local governments and residents, a deeper understanding of residents’ needs and preferences regarding public services can be achieved [27, 28]. This enhanced understanding enables local governments to effectively prioritize and allocate resources, ensuring they align accurately with residents’ demands. (2) fiscal decentralization empowers residents to evaluate and choose among local governments based on their performance. To attract and retain residents, local governments need to enhance public services and improve efficiency; Failing to do so puts them at risk of population decline and resource loss [29–32]. (3) the competition among local governments drives them to seek innovative ways to improve the quality and efficiency of public services [4, 33–35]. (4) the competition among local governments also necessitates the enhancement of information transparency and accountability mechanisms, enabling residents to supervise and evaluate government performance. As a result, local governments are compelled to make greater efforts to improve the efficiency of public services [36–39].

However, it is important to note that there are scholars who argue against the positive effects of local government competition on public service efficiency. Competition among local governments can lead to redundant and wasteful allocation of resources. In order to stand out in the competition, local governments may excessively invest resources, resulting in resource dispersion and inefficient utilization, which lowers the efficiency of public service [40–42]. Moreover, inter-governmental competition can give rise to negative externalities. Local governments, in their quest to attract talent and resources, may undertake actions that are detrimental to overall social welfare, such as lowering environmental standards or reducing labor rights protection [43, 44]. Additionally, fiscal decentralization can weaken the central government’s ability to invest in national infrastructure. When a significant portion of revenue and expenditure is assigned to local governments after decentralization, the central government may face challenges in carrying out equalizing transfer payments due to insufficient public resources [45, 46]. In addition, In China’s fiscal decentralization system, a third viewpoint suggests a non-linear impact of government competition on public service efficiency. Scholars like Ding argue that the growth effect of fiscal decentralization follows an inverted “U-shaped” curve. This is because the competition pressure in the “political tournament” differs for regions at different stages of development, leading to varying efforts from local governments [47]. Similarly, Chu employ the Malmquist-Luenberger (ML) productivity index to measure the efficiency of local government public service provision in China and confirm that fiscal decentralization has a nonlinear “inverted U-shaped” impact on the efficiency of education services [48].

### 2.3 Intergovernmental competition, market segmentation, and public service efficiency

Fiscal decentralization in China exhibits atypical characteristics [49]. Economic decentralization often coexists with political centralization, and the intense competition driven in the form of “GDP competition” among local governments is a prominent feature of fiscal decentralization in China [50]. The career advancement of local officials is closely tied to the economic performance of their regions, specifically measured by GDP growth rate and relative GDP ranking compared to neighboring provinces. In response to these political incentives, local officials may resort to implementing trade barriers or utilizing intangible preferential policies to protect vital industries within their local markets, thereby giving rise to market segmentation phenomena in China [5]. The impact of market segmentation on public service efficiency remains a topic of debate among scholars. Some argue that market segmentation can result in “fragmented economics” by narrowing market scope and causing overall economic inefficiency [51]. Others suggest that market segmentation hinders short-term economic growth [52]. However, research by Lu and Chen reveals an inverted U-shaped relationship between market segmentation and both short-term and future economic growth, indicating that market segmentation can promote economic growth in the short-term and future [53]. Jin also propose that that moderate fiscal decentralization effectively mitigates the distortions in factor allocation caused by market segmentation [54]. While there is no consensus on the impact of market segmentation on public service efficiency, it is widely recognized that market segmentation affects the efficiency of resource allocation [55–59] Liu emphasizes the importance of comprehensively addressing local protectionism and market segmentation issues in order to enhance China’s economic resource allocation efficiency, reduce regional and urban-rural disparities, and improve public service efficiency [60].

While there is a considerable body of literature on the impact of fiscal decentralization on public service efficiency, it is important to acknowledge certain limitations. (1) Many studies tend to rely on a single indicator to measure public service efficiency, overlooking the multidimensional nature of government service provision. Focusing solely on input or output indicators can lead to biased and inconsistent empirical results, as important explanatory variables may be omitted. (2) Most studies propose a single linear relationship between intergovernmental competition and public service efficiency under fiscal decentralization. However, in practice, this relationship is influenced by various factors, including a country’s political system, market mechanisms, and level of economic development. The relationship between government competition and public service efficiency continuously adjust with changes in internal and external factors, rendering a single linear relationship inadequate in accurately capturing the complex dynamics involved. (3) Existing research often discusses the impact of fiscal decentralization on local governments from a singular perspective. However, in China’s unique fiscal decentralization system, intergovernmental competition leads to market segmentation, which affects public resource allocation and subsequently influences public service efficiency. It is crucial to consider the implications of market segmentation resulting from intergovernmental competition on public service efficiency. (4) Previous literature suggests that inter-regional competition affects the efficiency of public service. Given the regional and temporal imbalances in China’s economic development, it is important to explore whether there is regional heterogeneity in the relationship between fiscal decentralization and public service efficiency. While Chen and Lai have measured differences in public service efficiency among regions, they did not specifically analyze the underlying reasons for these disparities within the context of fiscal decentralization [13]. Consequently, it becomes challenging to provide actionable recommendations for enhancing public service supply efficiency based on regional characteristics.

This article demonstrates innovation in in several aspects. Firstly, it introduces a comprehensive measurement of public service efficiency that considers both input and output dimensions, resulting in more precise assessments of public service efficiency. By incorporating a broader range of indicators, this approach provides a more comprehensive understanding of the effectiveness of government service provision.; Secondly, this article takes into account the phenomenon of market segmentation that accompanies fiscal decentralization. It integrates market segmentation, government competition, and public service efficiency into a unified analytical framework, revealing the non-linear effects of market segmentation and government competition on public service efficiency within the fiscal decentralization system. This contributes to complementing existing literature by providing additional perspectives. It helps in better understanding the relationship between public service efficiency and government competition, as well as market segmentation. Additionally, it sheds light on the factors that influence public service efficiency. Thirdly, the article explores the spatial interaction between public service provision and government competition, examining potential spillover effects on basic public services in neighboring areas. By considering the spatial dimension, the article provides valuable insights for optimizing spatial layout and informing policy decisions. Finally, the study employs a semi-parametric spatial lag panel model (SLSM), contrasting with previous studies that primarily used traditional panel regression or ordinary spatial panel regression models. The SLSM captures the relationship between market segmentation and public service efficiency through parameter estimation, while also accurately reflecting the impact of fiscal decentralization and inter-regional government competition on public service efficiency through non-parametric estimation. This modeling approach enhances the accuracy and reliability of the analysis. The remaining sections of this article are organized as follows. Section 3 describes the data, variables, and model construction. The baseline regression results and robustness tests are presented in Section 4. The conclusions and policy implications are discussed in Section 5.

## 3 Materials and methods

Chapter 3 is consist of four sections: data, variables, descriptive analysis of variables, and estimation methods. In Section 3.2, the selection process for explanatory variables, the dependent variable, and control variables is explained. The dependent variable, public service efficiency, is calculated using the DEA method. The explanatory variable, the market segmentation, is measured using the price index method. Other explanatory variable, fiscal decentralization, and interjurisdictional competition are chosen based on a review of the existing literature. Control variables are also sourced from relevant literature. In Section 3.4, the rationale for adopting the SLSM estimation method in this study is presented through 3D graphs, and then SLSM estimation method is introduced in detail.

### 3.1 Data

The data used in this study is panel data from 30 provinces in China from 2004 to 2021. Due to data availability, Tibet, Hong Kong, Taiwan, and Macao are not included in the analysis. The data on public service efficiency and market segmentation are sourced from the “China Statistical Yearbook (2005–2022)”. The indicators used to measure government competition are obtained from the Wind database, the annual “National Economic and Development Statistics Bulletin (2004–2021)” of the 30 provinces in China, and the “China Statistical Yearbook (2005–2022)”. The control variable data is sourced from the “National Economic and Social Statistics Bulletin (2004–2021)” and the “China Statistical Yearbook (2005–2022)” published by the National Bureau of Statistics of China, as well as the primary data from the seventh national census.

### 3.2 Variables

#### 3.2.1 Explained variable

The dependent variable in the model is Public Service Efficiency (PSE). Färe (1994) combined nonparametric linear programming methods with Data Envelopment Analysis (DEA) theory [61], which has led to the widespread application of the Malmquist index in measuring productivity efficiency across different sectors. In our study, the DEA-Malmquist model is used to measure public service efficiency.

The Malmquist index is a linear operation through the distance function, where the distance function is defined using the method of directional output and directional input, and the output distance function is defined as the optimal scale term of the output variable matrix given the input variable matrix. In this study, directed output variables are used to analyze the efficiency of public services in China, and the distance function of output variables is expressed as:

$$\begin{array}{c} D_{o}\left(x,y\right)=\text{inf}\left\{\delta :\left(x,y/\delta \right)\right\}\in p\left(x\right) \end{array}$$ (1)

Where the set of feasible output levels is denoted by *p*(*x*); *y* represents the output matrix, and *x* represents the input matrix.*δ* denotes the directed output efficiency index [62] (Fare, 1994b). If *y* lies within the interior of *p*(*x*);, the value of the distance function is less than 1; if *y* lies on the boundary of *p*(*x*); the value of the function will be equal to 1; and if *y* lies outside *p*(*x*); the value of the function will be greater than 1. For the change of overall efficiency from *t* to *t* + 1 under technical conditions in period *t*, the Malmquist index, which measures the growth of total factor productivity from the perspective of output, is written as:

$$\begin{array}{c} M\left(x^{t},y^{t},x^{t+1},y^{t+1}\right)={\left[\frac{D^{t}\left(x^{t+1},y^{t+1}\right)}{D^{t}\left(x^{t},y^{t}\right)}*\frac{D^{t+1}\left(x^{t+1},y^{t+1}\right)}{D^{t+1}\left(x^{t},y^{t}\right)}\right]}^{\frac{1}{2}} \end{array}$$ (2)

(*x*^*t*+1^, *y*^*t*+1^) (*x*^*t*^, *y*^*t*^) represent the input and output levels at time *t* + 1 and *t*. *D*^*t*^(*x*^*t*^, *y*^*t*^) is the distance function. According to Fare et al, the Malmquist index can be decomposed into three components: technological change (techch), pure efficiency change (peech), and scale efficiency change (sech) [62], which can be represented as:

$${\text{M}}^{t,t+1}=\frac{D_{v}^{t+1}(x^{t+1},y^{t+1})}{D_{v}^{t}(x^{t},y^{t})}*[\frac{D_{v}^{t}(x^{t},y^{t})}{D_{c}^{t}(x^{t},y^{t})}/\frac{D_{v}^{t+1}(x^{t+1},y^{t+1})}{D_{c}^{t+1}(x^{t+1},y^{t+1})}]*{\left[\frac{D_{c}^{t}(x^{t},y^{t})}{D_{c}^{t+1}(x^{t},y^{t})}*\frac{D_{c}^{t}(x^{t+1},y^{t+1})}{D_{c}^{t+1}(x^{t+1},y^{t+1})}\right]}^{\frac{1}{2}}$$ (3)

In our study, we considered 30 provinces in mainland China as decision-making units (DMUs), and the sample period is from 2004 to 2021. Tibet, Hong Kong, Taiwan, and Macao were excluded due to data unavailability. The sample period for our analysis spans from 2004 to 2021. To evaluate the input and output of public service efficiency, we referred to the studies of many scholars [63–65]. For the input indicators, we considered the proportion of local government financial expenditure allocated to education, social security and employment, healthcare and family planning, culture, sports, media, as well as urban-rural community expenses. These proportions were derived from the respective financial expenditure categories. Moreover, we selected commonly used output indicators, including the number of participants in end-of-year basic old-age insurance for urban and rural residents, the number of participants in end-of-year basic medical insurance, per capita annual expenditure on education, culture, and entertainment, the number of healthcare institutions per thousand people, the number of public buses and electric vehicles per ten thousand people, per capita park and green space area, and the number of public libraries. We chose these output indicators because their official provincial-level data can be directly collected from the “China Statistical Yearbook,” ensuring the reliability of our study. Using the DEAP2.1 software, we calculated statistical indices for public service efficiency.

#### 3.2.2 Explanatory variable

**Fig 2:**
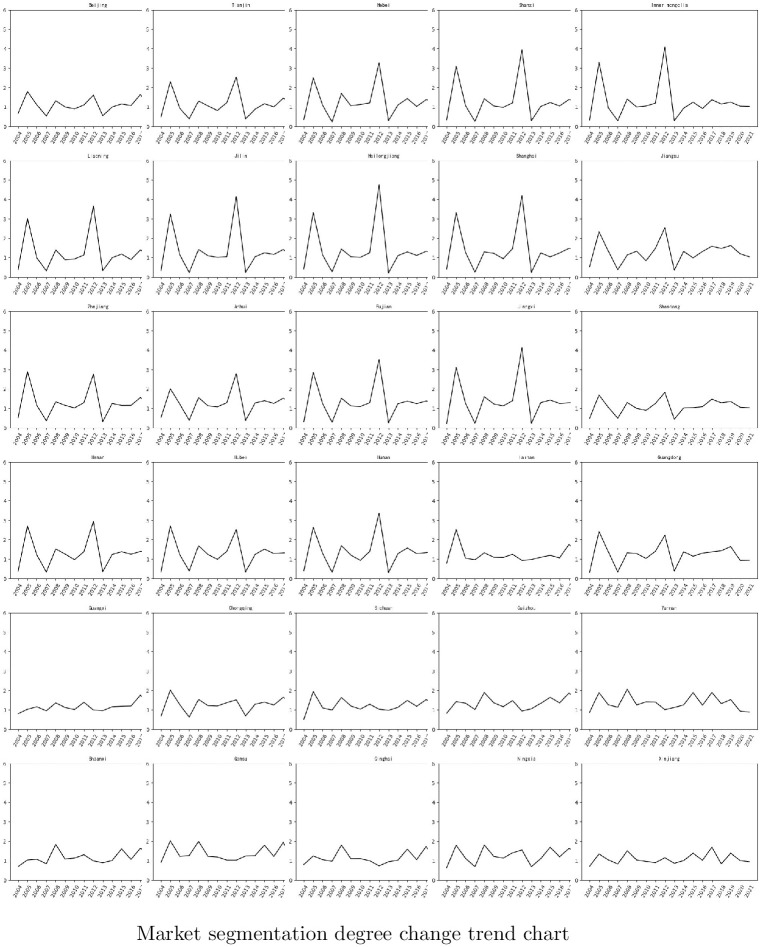


*(1) Market segmentation (SEG)*. This article uses the price method [66, 67] to measure market segmentation between 30 provinces in China (except Tibet, Macao, Taiwan, and Hong Kong) over 17 years from 2004 to 2021. To calculate the relative price differences of a set of commodities across regions over time, including the ones shown in Fig 2 three-dimensional panel data is needed (time, region, and commodity (*t* * *i* * *k*). The paper selects commodities based on Gui’s experience [68]. The process of computing the market segmentation index using the relative price method is as follows:

The first step involves calculating the absolute value $|\Delta Q_{ijt}^{k}|$ of the relative prices for 9 different commodities among adjacent provinces in China, $p_{it}^{k}$ is defined as the price of product *k* in province *i* at year *t*; the price difference between province *i* and *j* for a given product *k* at year *t* is as follows:

$$\begin{array}{c} \Delta Q_{ijt}^{k}=\text{ln}\left(\frac{p_{it}^{k}}{p_{jt}^{k}}\right)-\text{ln}\left(\frac{p_{it-1}^{k}}{p_{jt-1}^{k}}\right)=\text{ln}\left(\frac{p_{it}^{k}}{p_{it-1}^{k}}\right)-\text{ln}\left(\frac{p_{jt}^{k}}{p_{jt-1}^{k}}\right) \end{array}$$ (4)

A total of 66 pairs of adjacent provinces from 2004 to 2021 (a total of 17 years) resulted in 1122 differential forms of relative absolute price $|\Delta Q_{ijt}^{k}|$.

Then, market price differences caused by the characteristics of goods, we need to calculate the mean value of $|\Delta Q_{ijt}^{k}|$ to control for cross-sectional dependencies and then subtract the mean price difference from the $|\Delta Q_{ijt}^{k}|$, so, $q_{ijt}^{k}=|\Delta Q_{ijt}^{k}\left|-\right|\bar{\Delta Q_{ijt}^{k}}|$. The $q_{ijt}^{k}$ is the relative price change that is ultimately used to calculate the variance.

Thirdly, the variance of price changes for different commodities can be calculated as $var(q_{ijt}^{k})$, which yields the relative price change for the commodities. Finally, the market segmentation index for a province is obtained by averaging the index values of that province and all its neighboring provinces.

*(2) Fiscal Decentralization (FD)*. In practice, government competition takes two forms: vertical competition and interjurisdictional competition [69–74]. Vertical competition primarily occurs between the central and local governments. In this study, we follow the prevailing practice in the academic community, which measures competition between the central and local governments using the fiscal decentralization indicator. However, there is controversy surrounding how to measure the fiscal decentralization, and two main methods exist in the literature currently [75]. The first method is the fiscal revenue and expenditure indicator, which assesses the degree of fiscal decentralization based on the share of financial revenue and expenditure of the lower-level government. This method is predominantly used in China. However, when included in econometric models, this approach can lead to issues of multicollinearity. Therefore, we adopt the internationally accepted approach, which calculates the ratio of per capita local government expenditures to the sum of per capita local government expenditures and per capita central government expenditures. This method avoids the problem of multicollinearity and provides a more robust measure of fiscal decentralization for our analysis.

*(3) Interjurisdictional competition (IC)*. Interjurisdictional competition of the government pertains to the relationships between local governments. There is no universally recognized measurement indicator for this type of competition. In line with the approach taken by Fu and Zhang [76], this study employs the actual foreign direct investment (FDI) amounts in each region as the explanatory variable, and the level of foreign direct investment is measured by dividing the total yuan value into GDP and excluding the price factor. Additionally, we take into account the annual average exchange rate of the US dollar for the respective year. This ensures that the measurement of foreign direct investment is accurate and consistent across regions and over time [77].

#### 3.2.3 Control variables

*(1) Economic development (ECO)*. Referring to previous relevant studies, this paper selects per capita GDP; higher levels of regional economic development can enhance residents’ demand for public services and their political influence, which may encourage the government to improve the efficiency of public service supply. On the other hand, an increase in income level also means an increase in the cost of public service supply, resulting in a potential decrease in local government public service supply efficiency. Therefore, the final impact of per capita GDP on the efficiency of local government’s basic public service supply is uncertain.

*(2) Industry structure (IND)*. The industry structure is measured by the ratio of the output value of secondary and tertiary industries [50].

*(3) Level of education (EDU)*. It is measured by years of schooling per capita in each province. Improvements in residents’ education levels can enhance their sense of democratic participation and ability to participate [78], which may ultimately urge local governments to improve the efficiency of public service supply. Similar conclusions have been supported by international empirical studies [79]. However, the impact of education on public service supply efficiency in China remains uncertain due to the country’s top-down administrative management system and weak political influence of residents, making it difficult for such an influence mechanism to emerge [80].

*(4) Degree of opening-up (OPN)*. The ratio of total imports of each province (by domestic destination and source) to its gross domestic product (RMB).

*(5) Population density (POP)*. We use the logarithmic form of population per square kilometer to measure population density.

*(6) Technology innovation (TEC)*. Technology innovation is measured by the logarithmic form of the number of patents granted by each province.

### 3.3 Descriptive statistics

Table 1 shows the descriptive statistical analysis of relevant data in this paper.

**Table 1:** The variables description.

Variable	Code	N	Mean	Sd.	Min	Max
y	PSE	540	1.248	0.621	0.205	4.748
Wy	W.PSE	540	1.243	0.232	0.618	2.807
X1	SEG	540	0.000	0.000	0.000	0.002
X2	FD	540	0.829	0.068	0.599	0.987
X3	IC	540	0.021	0.020	0.000	0.121
X4	ECO	540	10.474	0.711	8.346	12.123
X5	IND	540	1.100	0.631	0.494	5.297
X6	EDU	540	8.863	1.037	6.378	12.782
X7	OPN	540	0.293	0.335	0.007	1.876
X8	POP	540	5.439	1.269	2.010	8.275
X9	TEC	540	9.476	1.724	4.248	13.679

### 3.4 Research model

**Fig 3:**
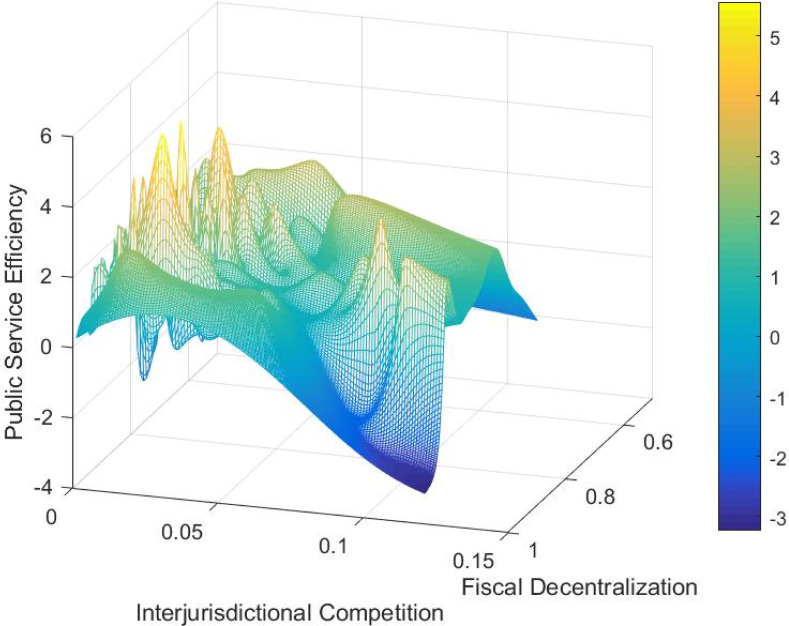


Fig 3 illustrates the impact of changes in fiscal decentralization and intergovernmental competition on public service efficiency. The relationship between fiscal decentralization and public service efficiency is represented by an inverse “U” shape on the graph, indicating that the positive influence of fiscal decentralization on public service efficiency eventually diminishes and turns negative.Similarly, the relationship between intergovernmental competition and public service efficiency is depicted by an “M” shape, suggesting that the impact of local government competition on public service efficiency fluctuates. As shown in the Fig 3, governmental competition, including fiscal decentralization and intergovernmental competition, can have a nonlinear impact on local government public service efficiency. In the context of market segmentation, public service efficiency exhibits spatial heterogeneity. Therefore, it is necessary to consider employing spatial econometric models to analyze the spatial heterogeneity and nonlinear relationship between market segmentation, governmental competition, and public service efficiency of local governments in China. The nonparametric term of the semi-parametric model effectively avoids the specification error caused by linear relationship assumptions in parametric models, while also converging faster than nonparametric models. Therefore, this paper introduces a semi-parametric spatial lag panel model (SLSM) in analyzing the effects of governmental competition and market segmentation on public service efficiency in China. The model is specified as follows:

$$\begin{array}{c} PSE_{it}=\alpha_{i}+\rho W_{ij}PSE_{jt}+\beta_{1}SEG_{it}+\sum_{k=2}^{7}\beta_{k}control_{k}+G\left(Z_{it}\right)+\varepsilon_{it} \end{array}$$ (5)

$$\begin{array}{c} PSE_{it}=\alpha_{i}+\rho W_{ij}PSE_{jt}+\beta_{1}SEG_{it}+\sum_{k=2}^{7}\beta_{k}control_{k}+G\left(Q_{it}\right)+\varepsilon_{it} \end{array}$$ (6)

*PSE*_*it*_ represents the explained variable; *ρ* is the coefficient of the spatial lag term of the dependent variable;*SEG*_*it*_ represents market segmentation as one of the core explanatory variables; *Control*_*k*_ is a set of control variables, *β* is the coefficient of the explanatory variable, and *G*(.) indicates the unknown non-parametric part. *Z*, *Q* represents the explanatory variables of FD and IC as the non-parametric component; *ε* is the residual term. The space weight matrix represented by *W*_*ij*_. The equation is as follows:

$$\begin{array}{c} W_{ij}=\{\begin{array}{c} 1/d_{ij},\;i\neq j\\ 0,\;i=j \end{array} \end{array}$$ (7)

In the equation, *d*_*ij*_ represents the distance between the spatial cross section of provinces *i* and *j*. To estimate Eq 5, the parametric components *ρ*, *β*_1_, and *β*_*k*_, as well as the nonparametric component *G*(.), are estimated using the following method: Firstly, assuming that the parameters *ρ*, *β*_1_, *β*_*k*_ is known, Eq 8 can be obtained from Eq 5:

$$\begin{array}{c} \begin{array}{c} G\left(Z_{it}\right)=E\left(PSE_{it}|Z_{it}\right)-\alpha_{i}-\rho E\left(W_{ij}PSE_{jt}|Z_{it}\right)\\ \;-\beta_{1}\left[E\left(SEG_{it}|Z_{it}\right)\right]-\beta_{k}\sum_{k=2}^{7}E\left(control_{k}|Z_{it}\right) \end{array} \end{array}$$ (8)

Thus, a preliminary estimate of the nonparametric component *G*(.) is obtained:

$$\begin{array}{c} \begin{array}{c} \hat{(}Z_{it};\rho ,\beta_{1},\beta_{k})=\hat{E}\left(PSE_{it}|Z_{it}\right)-\alpha_{i}-\rho \hat{E}\left(W_{ij}PSE_{jt}|Z_{it}\right)\\ \;-\beta \left[\hat{E}\left(SEG_{it}|Z_{it}\right)\right]-\beta_{k}\sum_{k=2}^{7}\hat{E}\left(control_{k}|Z_{it}\right) \end{array} \end{array}$$ (9)

Substituting the preliminary estimate of the nonparametric component into Eq 9, we obtain the following parametric model for eliminating *α*_*i*_:

$$\begin{array}{c} \begin{array}{c} PSE_{it}-\hat{E}\left(PSE_{ij}\right)|Z_{it})=\rho [W_{ij}PSE_{jt}-\hat{E}\left(W_{ij}PSE_{jt}|Z_{it}\right]+\beta_{1}[SEG_{it}-\\ \hat{E}\left(SEG_{it}|Z_{it}\right)]+\beta_{k}\sum_{k=2}^{7}\left[control_{k}-\hat{E}\left(control_{k}|Z_{it}\right)\right]+\varepsilon_{it} \end{array} \end{array}$$ (10)

Then the tool variable is used for two-stage least squares estimation, and the estimation $\hat{\rho }$, ${\hat{\beta }}_{1}$, ${\hat{\beta }}_{k}$ of parameter *ρ*, *β*_1_, *β*_*k*_ is obtained. The resulting estimates are then used to obtain the estimated nonparametric component $\hat{G}\left(Z_{i}t\right)$ and its first partial derivative ∂*G*(.)/∂*Z*_*it*_

$$\begin{array}{c} \hat{G}\left(Z_{it}\right)=\hat{G}\left(Z_{it};\rho ,\beta_{1},\beta_{k}\right) \end{array}$$ (11)

$$\begin{array}{c} \frac{\partial G(.)}{\partial Z_{it}}=\frac{\hat{G}\left(Z_{it};\rho ,\beta_{1},\beta_{k}\right)}{\partial Z_{it}} \end{array}$$ (12)

In general, we have constructed a semi-parametric panel spatial lag model (SLSM) consisting of two main components: the parametric part with SEG as the explanatory variable, and the non-parametric part with FD and IC as the explanatory variables. The non-parametric component involves an unknown nonlinear function G that captures the relationship between FD and PSE, as well as IC and PSE. To estimate the non-parametric part, we employ the methods outlined in Eqs 8–12. Subsequently, we utilize a two-stage least squares estimation with instrumental variables to obtain the parameter estimates. The estimation method for Eq 6 is consistent with that of Eq 5.

## 4 Empirical analysis

Section 4 consists of four parts. To ensure the reliability of the data, this study conducts unit root tests and cointegration tests in 4.1. In 4.2, spatial autocorrelation analysis is employed to validate the spatial variations in public service efficiency. Three estimation methods, namely OLS, SLM, and SLSM, are applied in 4.3 to examine the impact of market segmentation and government competition on public service efficiency. In 4.4, robustness tests are conducted by replacing variables and excluding specific years to validate the results.

### 4.1 Panel unit root and cointegration test

The study employed three methods, namely the LLC test, the ADF-fisher test, and the IPS test, to examine the presence of unit roots and ensure reliable regression modeling. The results, presented in Table 2, indicate that the original sequences of PSE, SEG, FD, and EDU are stationary under all three test methods, while IC, ECO, IND, OPN, POP, and TEC are found to be first-order integrated sequences. Furthermore, the unit root tests demonstrate that both the dependent and independent variables satisfy the conditions of first-order cointegration. To test for cointegration, we applied the panel cointegration test proposed by Peter Pedroni [81] and the results are displayed in Table 3. All statistics are significant at the 1% significance level, rejecting the null hypothesis of no cointegration relationship between the first-order integrated variables. This suggests that all variables included in the regression model exhibit a long-term cointegration relationship and can be directly used for modeling purposes.

**Table 2:** Unit root test.

	Level			1st difference		
Variable	LLC	ADF	IPS	LLC	ADF	IPS
PSE	-21.9136***	328.3736***	-4.9672***	-37.3201***	605.8000***	-24.7093***
	(0.0000)	(0.0000)	(0.0000)	(0.0000)	(0.0000)	(0.0000)
SEG	-19.2472***	248.3829***	-4.3549***	-41.6151***	494.6308***	-26.4541***
	(0.0000)	(0.0000)	(0.0000)	(0.0000)	(0.0000)	(0.0000)
FD	-9.0435***	99.2854**	-2.0514**	-23.1271***	183.4221***	-11.6156***
	(0.0010)	(0.0011)	(0.0117)	(0.0000)	(0.0000)	(0.0000)
IC	-12.2490***	71.2119	-1.9976***	-23.8418***	231.9968***	-11.7043***
	(0.0000)	(0.1525)	(0.0306)	(0.0000)	(0.0000)	(0.0000)
ECO	-10.2273***	51.0840	-2.4969***	-23.6750***	175.7746***	-12.4021***
	(0.0010)	(0.7871)	(0.0000)	(0.0000)	(0.0000)	(0.0000)
IND	-2.3084**	49.66921	-2.3084**	-24.4402***	174.8381***	-11.3324***
	(0.0020)	(0.8268)	(0.0020)	(0.0000)	(0.0000)	(0.0000)
EDU	-16.8444***	128.6236***	-3.3743***	-28.6523***	397.1872***	-16.6215***
	(0.0000)	(0.0000)	(0.0000)	(0.0000)	(0.0000)	(0.0000)
OPN	-14.0641***	65.6231	-2.6788***	-26.6715***	271.3349***	-12.7769***
	(0.0000)	(0.2882)	(0.0000)	(0.0000)	(0.0000)	(0.0000)
POP	-3.2957	36.1845	-1.9226	-29.5514***	97.9857**	-14.9071***
	(0.9995)	(0.9936)	(0.5413)	(0.0000)	(0.0014)	(0.0000)
TEC	-10.9726***	49.7804	-1.3666	-21.2417***	125.3805**	-9.1565
	(0.0000)	(0.8238)	(0.7638)	(0.0001)	(0.0000)	(0.0000)

***p<0.01,

**p<0.05,

*p<0.1.

**Table 3:** Cointegration test.

	Statistic	p-value
Modified Phillips–Perron t	8.3142	0.0000
Phillips–Perron t	-10.1196	0.0000
Augmented Dickey–Fuller t	-5.3547	0.0000

### 4.2 Spatial correlation analysis of public service efficiency

According to the findings presented in Table 4, the Moran’s I index of Chinese local government public service efficiency supports our hypothesis at the 1% significance level, with the exception of 2020 and 2021. This indicates a significant presence of spatial autocorrelation in market segmentation across the 30 provinces of China. This spatial autocorrelation can be attributed to the potential spillover effect of market segmentation and government competition on the efficiency of public services. In other words, the efficiency of local public services can be influenced by market segmentation and government competition in other locations.

**Table 4:** Moran’s I of PSE in China from 2004 to 2021.

**Year**	**2004**	**2005**	**2006**	**2007**	**2008**	**2009**	**2010**	**2011**	**2012**
Moran’s I	0.339***	0.261***	0.253***	0.460***	0.311***	0.274***	0.224***	0.313***	0.407***
**Year**	**2013**	**2014**	**2015**	**2016**	**2017**	**2018**	**2019**	**2020**	**2021**
Moran’s I	0.474***	0.417***	0.322***	0.305***	0.332***	0.227***	0.348***	-0.000***	-0.095***

***, **, and * denote statistical significance levels at 1%, 5%, and 10%, respectively (similarly, hereinafter)

**Fig 4:**
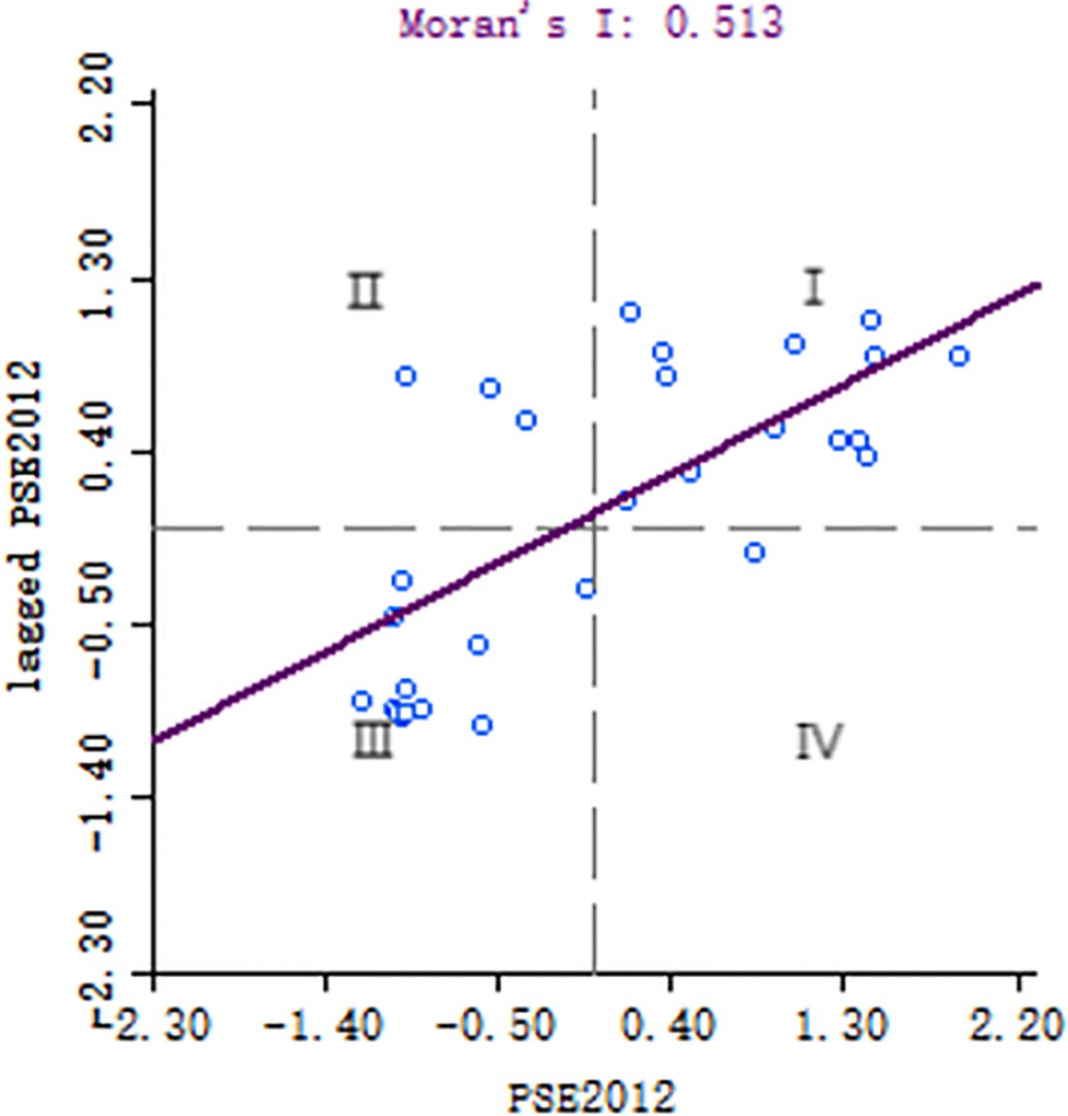


**Fig 5:**
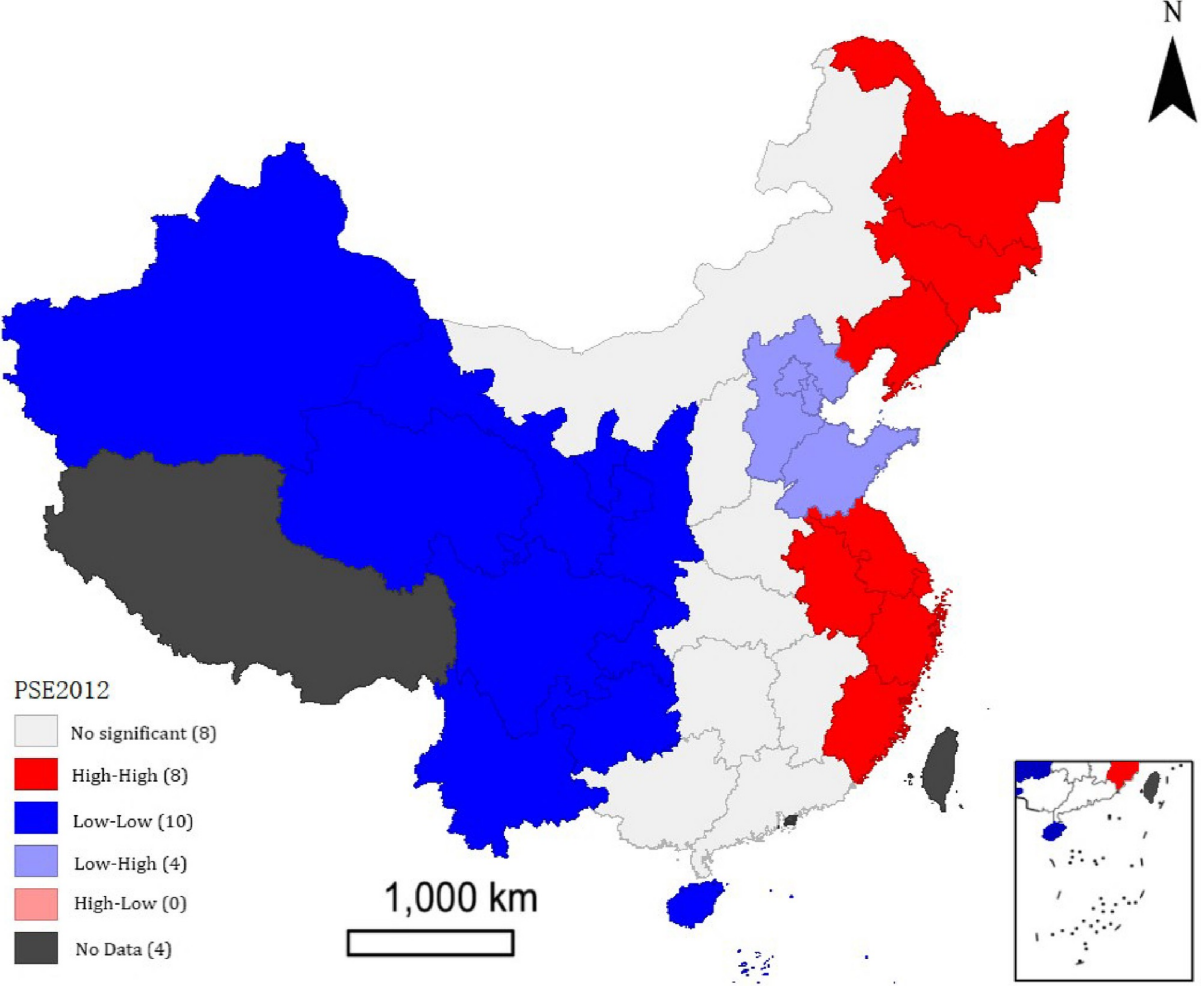


This paper also analyzed the local spatial auto-correlation of public service efficiency. Fig 4 displays the Moran scatter chart, while Fig 5 presents the LISA chart, both depicting Spatial correlation of public service efficiency in provincial local governments in 2012. The Moran scatter plot, represented by the Moran’s I index, visualizes the relationship between the spatial lag factor and the public service efficiency in a two-dimensional plot. Quadrant 1 represents regional units with high public service efficiency surrounded by other high-value regions (HH). Quadrant 3 represents regional units with low public service efficiency enclosed by other low-value areas (LL). Quadrants 2 and 4 indicate negative spatial correlation. Quadrant 2 shows regional units with low public service efficiency surrounded by high-value regions (LH), while quadrant 4 represents areas with high public service efficiency surrounded by low-value regions (HL). In 2012, the Moran’s I scatter diagram of China’s public service efficiency exhibited a standardized Moran’s I index. Most areas were located in quadrants 1 and 3, indicating a positive correlation of regional public service efficiency. The spatial distribution of public service efficiency demonstrated notable differences between the eastern and western regions of China. The concentration of high public service efficiency in Jiangsu, Shanghai, Anhui, Zhejiang, and Fujian can be attributed to their developed economies and spatial clustering. On the other hand, the low concentration of public service efficiency in Xinjiang, Qinghai, Gansu, Sichuan, and Yunnan can be attributed to relatively scarce resources, capital, and technology in those regions. Furthermore, to explain the phenomenon, the paper employs the semi-parametric spatial lag panel model (SLSM) at the provincial level in China.

### 4.3 Benchmark regression analysis

Linear regression model (OLS), spatial lag model (SLM), and semi-parametric spatial panel lag model (SLSM) were used to investigate the impact of market segmentation and government competition on the efficiency of public services. The data utilized in the analysis can be found in S1 File, and the results are compared in Table 6. The results obtained from the linear regression model indicate that, with the exception of population density, none of the factors examined have a significant impact on local government public service efficiency. This finding is in clear contradiction to both theoretical expectations and real-world observations.

To address this, Lagrange Multiplier (LM) (Table 5) tests were conducted, followed by the introduction of a fixed-effects spatial panel lag model. The regression results of this model demonstrate that market segmentation and population density have a significant impact on public service efficiency, which aligns with the viewpoints of Grossman [19], Zhang [82], Wang [83], and Chen [84]. However, the impact of intergovernmental competition on public service efficiency is not found to be statistically significant. Does this imply that there is no relationship between intergovernmental competition, fiscal decentralization, and public service efficiency? It is possible that the non-linear relationship between PSE and FD, IC may account for this issue.

**Table 5:** LM test.

Test	Statistic	df	p-value
Spatial error:			
Moran’s I	31.817	1	0.000
Lagrange multiplier	966.063	1	0.000
Robust Lagrange multiplier	1.648	1	0.199
Spatial error:			
Lagrange multiplier	967.459	1	0.000
Robust Lagrange multiplier	3.044	1	0.081

To further refine the analysis, a semi-parametric spatial lag model is employed. The estimation of the non-parametric component is carried out using the Epanechnikov kernel function and fixed-window width local linear instrumental variable estimation. This estimation procedure is implemented using both Matlab and Stata software. The results of the semi-parametric spatial lag model are presented in Table 6. It can be observed that the R^2^ value of the semi-parametric spatial lag model is significantly higher than that of the previous two regression models. This indicates a superior goodness of fit and a better ability to explain the impact of various variables on public service efficiency. The semi-parametric spatial lag model provides a more comprehensive and accurate understanding of the relationships between the variables and public service efficiency.

**Table 6:** Regression results of three models.

	OLS	SLM			SLSM	
		ind	time	both	model(5)	model(6)
SEG	-233.687	-143.296*	-232.576**	-208.965**	-322.828*	-333.087*
	(190.038)	(85.529)	(100.268)	(100.838)	(193.899)	(193.345)
FD	1.153	-0.156	0.569	-0.032		
	(0.885)	(0.594)	(0.472)	(0.774)		
IC	-0.551	0.706	-0.053	0.630		
	(1.151)	(1.089)	(0.699)	(1.150)		
ECO	0.005	-0.010	-0.091	-0.027	0.144	0.213
	(0.124)	(0.074)	(0.063)	(0.088)	(0.202)	(0.188)
IND	-0.018	-0.045	-0.034	-0.065	0.288	0.136
	(0.059)	(0.053)	(0.029)	(0.064)	(0.279)	(0.280)
EDU	-0.083	-0.046	0.005	-0.009	-0.333**	-0.300**
	(0.056)	(0.056)	(0.027)	(0.075)	(0.150)	(0.140)
OPN	-0.075	-0.030	-0.006	-0.040	-0.553	-0.842
	(0.109)	(0.137)	(0.065)	(0.149)	(0.710)	(0.704)
POP	0.072*	0.118	0.040**	0.157	0.959	0.253
	(0.038)	(0.300)	(0.018)	(0.320)	(1.673)	(1.583)
TEC	-0.018	0.030	-0.007	0.061	-0.281*	-0.111
	(0.034)	(0.037)	(0.017)	(0.052)	(0.155)	(0.145)
Intercept	0.855					
	(0.598)					
W.PSE					-1.946***	-0.021***
					(0.104)	(0.103)
N	540	540	540	540	510	510
R^2^	0.0146	0.0004	0.0006	0.0001	0.4630	0.4718

***, **, and * denote statistical significance levels at 1%, 5%, and 10%, respectively (similarly, hereinafter)

#### 4.3.1 The spatial effect of public service efficiency is statistically significant

**Fig 6:**
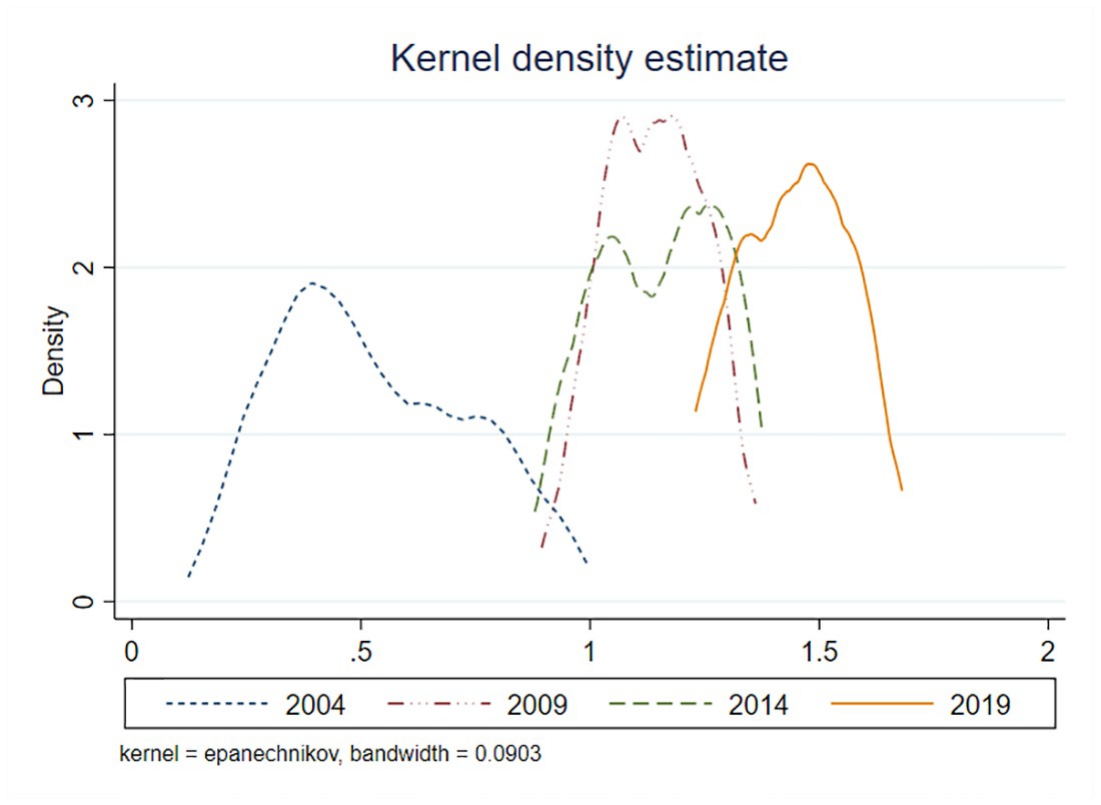


This study examines the regional disparities in public service efficiency in China by calculating the global Moran’s I index. Table 4 presents positive values for all years, except for 2020 and 2021, which pass the significance test. This suggests a significant positive spatial correlation in the development of public services, indicating spatial agglomeration throughout the observation period. Further analysis is conducted using local spatial autocorrelation tests specifically for public service efficiency in 2012. The results reveal significant variations in spatial clustering of public service efficiency between eastern and western regions of China. The eastern coastal regions, such as Jiangsu, Shanghai, Anhui, Zhejiang, and Fujian, exhibit significant high-high agglomeration of public service efficiency. This can be attributed to their higher economic levels, well-connected infrastructure, and spatial clustering of public services resources. Conversely, the western regions including Xinjiang, Qinghai, Gansu, Sichuan, and Yunnan show significant low-low agglomeration of public service efficiency due to relatively limited resources, funding, and technology, resulting in an overall lag in public service efficiency. To further verify the dynamic evolution of public service efficiency, kernel density estimation is applied to explore the distribution characteristics of national public service development, as depicted in Fig 6. The distribution curve exhibits a rightward shift, indicating an overall improvement in China’s public service level during the observation period. The distribution curve demonstrates a rightward shift, with a noticeable increase in the height of the main peak and a reduction in overall curve width. The distribution curve exhibits a rightward shift, indicating an overall improvement in China’s public service level during the observation period. The increase in the height of the main peak and the reduction in overall curve width further support this trend. The high estimated values corresponding to the main peak indicate a continuous enhancement of public service efficiency, along with a narrowing of regional disparities. The curve demonstrates a bimodal distribution in 2009 and 2014, suggesting a polarization of public services in recent years. However, over time, the bimodal distribution gradually transitions to a weaker unimodal pattern, indicating a diminishing polarization in public services. Overall, the narrowing distribution curve reflects a convergence of public service efficiency nationwide, indicating a trend towards greater consistency in public service efficiency across China.

#### 4.3.2 Market segmentation inhibits the efficiency of public services

The regression results of Model 1 and Model 2 confirm that market segmentation inhibits the efficiency of government public services. To more accurately capture the relationship between market segmentation and public service efficiency efficiency, the control variables were removed and a regression of public service against market segmentation was conducted. The results, presented in column (1) of Table 7, still indicate a significant negative effect of market segmentation on public service efficiency. In addition,from a regional perspective, there are significant differences in the impact of market segmentation on public service efficiency across China’s western, central, and eastern regions. As depicted in columns (2)-(4) of Table 7, market segmentation in the western region demonstrates a significant positive influence on public service efficiency. This can be attributed to the overall economic backwardness and inadequate market development in the western region. When local governments implement market segmentation policies that protect local enterprises and restrict external competition, it is more conducive to local economic development and the improvement of public service levels. On the other hand, in economically developed regions such as eastern China, maintaining property rights and markets, encouraging competition, expanding regional integration, and reducing market segmentation are more favorable for promoting local economic development. The influence of market segmentation on public service efficiency in China’s central region is not significant, mainly due to the significant differences in economic development levels within this region, making it difficult to accurately assess the true impact of market segmentation on public service efficiency. These findings are supported by Xu and Fang, who argue that the choice between market segmentation and regional integration depends on the trade-offs made by local government officials to achieve political promotion. When he efforts of local governments have positive spillover effects, regional integration is preferred rationally. Conversely, market segmentation between regions becomes the optimal choice for local governments. Specifically, implementing market segmentation is advantageous for backward regions in the early stages of economic development, while encouraging regional integration is more beneficial for economically advanced regions [85, 86].

**Table 7:** Regional regression results.

	(1)	(2)	(3)	(4)
SEG	-230.851***	405.850**	-375.197	-219.985**
		(173.596)	(1173.029)	(0.075)
FD				
IC				
ECO		0.084	0.067	-0.003
		(0.113)	(0.401)	(0.080)
EDU		-0.008	0.352	-0.110***
		(0.057)	(0.216)	(0.033)
OPN		-0.092	-0.599	0.170
		(0.098)	(1.616)	(0.207)
POP		-0.035	0.292	0.045***
		(0.067)	(0.416)	(0.015)
TEC		0.007	0.077	-0.015
		(0.026)	(0.122)	(0.016)
N	540	198	162	180
R^2^	0.002	0.001	0.030	0.054

***, **, and * denote statistical significance levels at 1%, 5%, and 10%, respectively (similarly, hereinafter)

#### 4.3.3 Government competition has a nonlinear impact on public service efficiency

**Fig 7:**
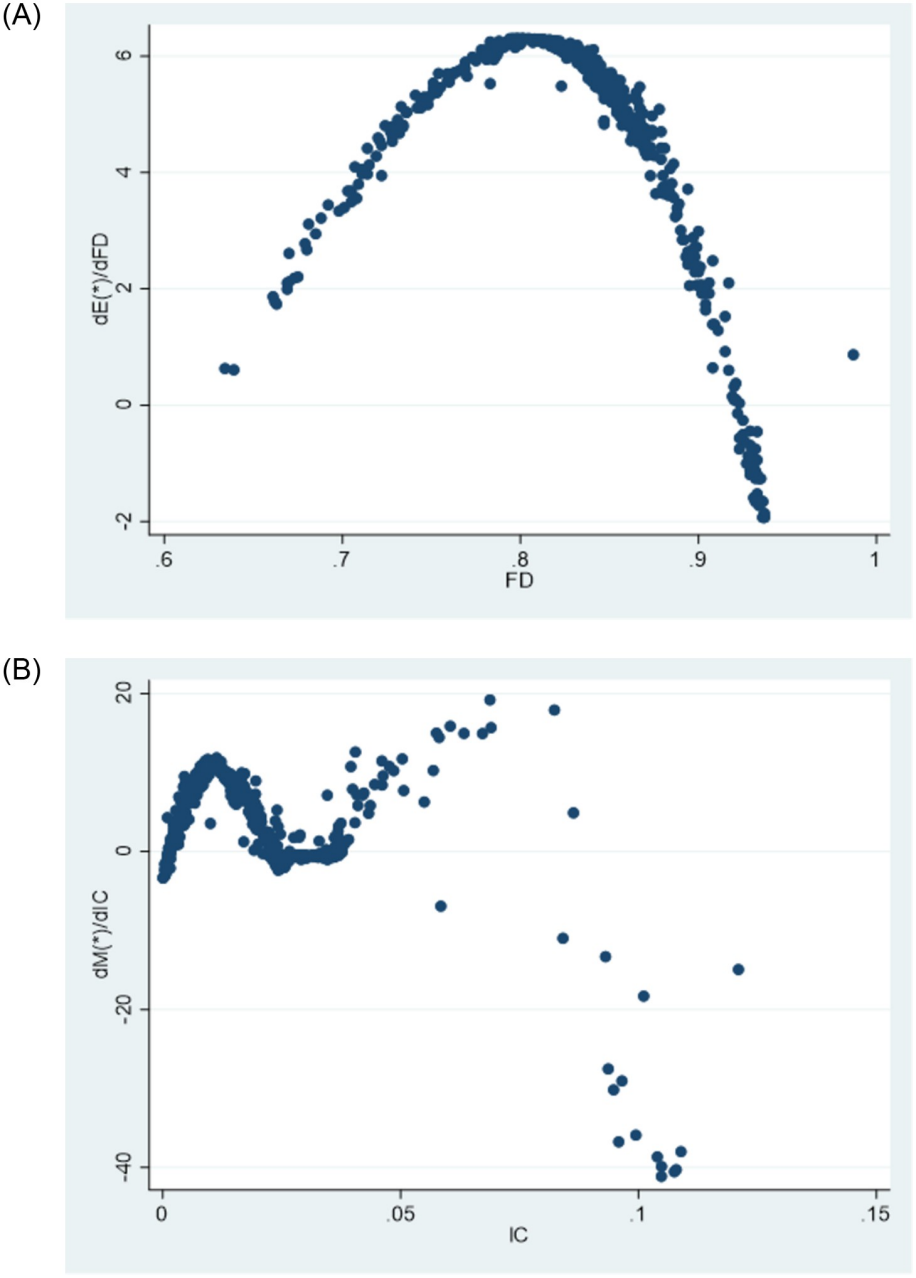


The impact of government competition on public service efficiency has been analyzed in our econometric model from two perspectives: vertical competition, measured by fiscal decentralization (FD) in model 1, and intergovernmental competition (IC), measured by per capita FDI decentralization in model 2, The measurement results are displayed in Fig 7, with the left side representing the partial derivative plot of model 1 and the right side representing the measurement results of model 2. The partial derivative plot of model 1 (Fig 7, left) visually reflects the nonlinear effect of vertical competition on public service efficiency. The horizontal axis represents fiscal decentralization, while the vertical axis represents the partial derivative of public service efficiency, indicating the incremental impact of a 1% increase in fiscal decentralization on public service efficiency. The scatter plot exhibits an inverted U-shape. From the scatter plot, several observations can be made: (1) When the degree of fiscal decentralization is less than 0.808, vertical competition has a promoting effect on public service efficiency. This can be attributed to the influence of fiscal incentives, constraints, and public supervision on local government behavior within a fiscal decentralization system. Under such conditions, officials are guided to invest in public welfare, reduce corruption and rent-seeking, optimize the allocation of public resources, and enhance the efficiency of public service provision. The competitive dynamics resulting from moderate fiscal decentralization further stimulate improvements in local public service provision efficiency [65]; (2) However, as fiscal decentralization exceeds 0.808, the partial derivative of fiscal decentralization on public service efficiency starts to decrease, and it may even become negative. This can be attributed to the marginal effects of fiscal decentralization on public service efficiency. Although theoretically fiscal decentralization is expected to continuously release the incentive effect on local government provision of public services with increasing decentralization, in practice, the incentive effect diminishes due to the scale inefficiency resulting from differentiated regional public service provision, rent-seeking behavior of local government officials, and corruption. As pointed out by Keen and Marchand, competition among local governments under fiscal decentralization can lead to “deviations” in the structure of public expenditure, resulting in systematic distortions [36]. Additionally, the inverted U-shaped effect of fiscal decentralization on public service efficiency suggests the existence of an optimal level of fiscal decentralization in China. Unfortunately, due to space limitations, further discussion on this topic is beyond the scope of this paper.

Fig 7 (right) shows the partial derivative plot of intergovernmental competition on public service efficiency, reflecting the oscillating marginal impact of intergovernmental competition on the incremental effect of public service efficiency. This non-linear relationship further supports the validity of the non-parametric specification. This phenomenon can be attributed to the dual effects of competition among local governments on public service efficiency. On one hand, intergovernmental competition among local officials for “political achievements” drives improvements in local infrastructure, attracting investments and promoting local economic growth. This helps enhance local public service facilities, improve service quality, and increase service efficiency. On the other hand, in the pursuit of victory in regional competition, local governments may adopt protective and predatory strategies, resulting in inefficient resource allocation and undermining the improvement of public service efficiency. The direction of the impact of horizontal government competition on public service efficiency depends on the relative strengths of these two effects. When the negative effect outweighs the positive effect, an increase in the level of horizontal competition among local governments leads to a decrease in public service efficiency. Conversely, when the positive effect outweighs the negative effect, an increase in the level of horizontal competition leads to an improvement in public service efficiency. This conclusion substantiates the previous hypothesis and demonstrates the dynamic nature of the influence of government competition on public service efficiency. It also helps explain the divergent views held by scholars on this issue.

#### 4.3.4 The impact of other factors on public service efficiency

The finding of a significant negative relationship between the education level of residents and public service efficiency may be surprising. However, it aligns with the earlier proposition in the paper, which suggests that under the institutional framework of political centralization in China, local officials are appointed by higher-level authorities. This makes it unlikely for the education level of residents to have a positive incentive mechanism to influence local officials in improving government expenditure efficiency through processes such as elections and oversight [76]. The lack of significant influence from factors such as economic development and openness to the outside world on public service efficiency could be attributed to the existing administrative system in China. In this system, the provision and management of public services are primarily carried out by government institutions, and the allocation of resources and improvement of public service efficiency are heavily reliant on government intervention. As a result, factors such as economic development, industry structure and degree of opening-up have a limited direct impact on public service efficiency. Additionally, the weak negative influence of science and technology on public service efficiency could be explained by the spatial clustering of technological services and the presence of siphon effect. The concentration of technological services in certain areas may hinder the improvement of innovation levels in neighboring regions, thereby affecting public service efficiency.

### 4.4 Robustness test

#### 4.4.1 Indicator replacement

**Fig 8:**
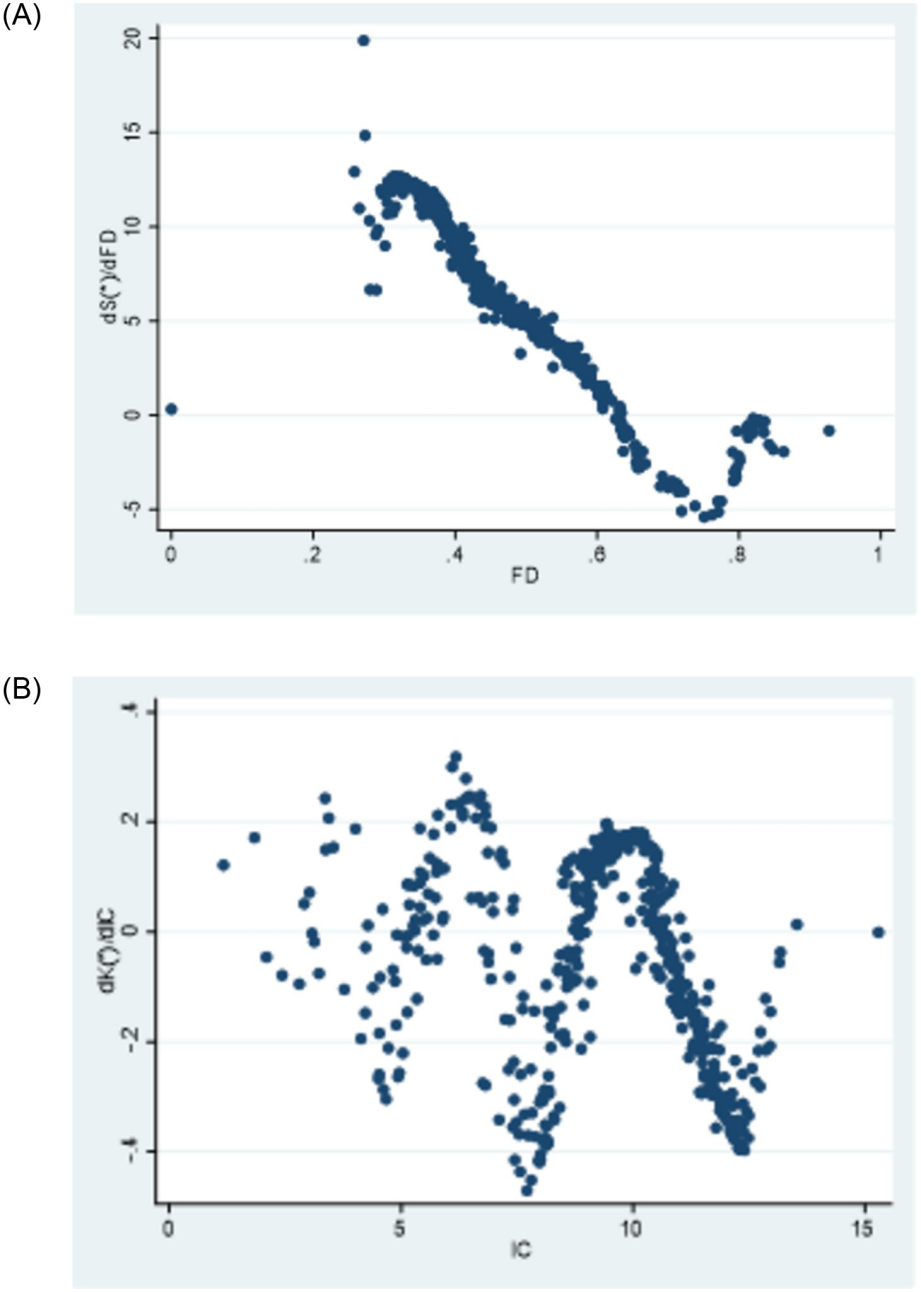


To ensure the robustness of the regression results, we conducted sensitivity tests by replacing the core explanatory variables. Specifically, we used fiscal revenue decentralization as a proxy variable for fiscal decentralization in the original model. Additionally, we utilized the sub-index “development of non-state economy” from the Marketization Index as a proxy indicator for horizontal government competition. Similar to the previous section, we employed a semi-parametric spatial lag regression and generated partial derivative plots for the non-parametric component. The results are presented in Table 8, and Fig 8. Table 8 indicates that the regression coefficient for market segmentation is consistent in sign and statistically significant. The partial derivative plot for intergovernmental competition aligns with the previous findings, exhibiting oscillatory patterns. The partial derivative plot for fiscal decentralization shows slight differences compared to the previous results, but overall, the effect of fiscal decentralization on public service efficiency changes from positive to negative, consistent with the overall trend observed earlier. This indicates the robustness of the results.

**Table 8:** Robustness test result (1).

	(1)	(2)
	PSE	PSE
W.PSE	-1.981***	-1.916***
	(0.101)	(0.102)
SEG	-372.603*	368.570*
	(191.546)	(191.876)
ECO	0.283	0.089
	(0.189)	(0.190)
IND	0.226	0.279
	(0.281)	(0.277)
EDU	-0.289**	-0.311**
	(0.141)	(0.140)
OPN	-0.479	-0.440
	(0.719)	(0.697)
POP	0.115	2.154
	(1.603)	(1.649)
TEC	-0.109	-0.333**
	(0.157)	(0.148)
N	510	510
R^2^	0.4728	0.4798

#### 4.4.2 Remove special years

**Fig 9:**
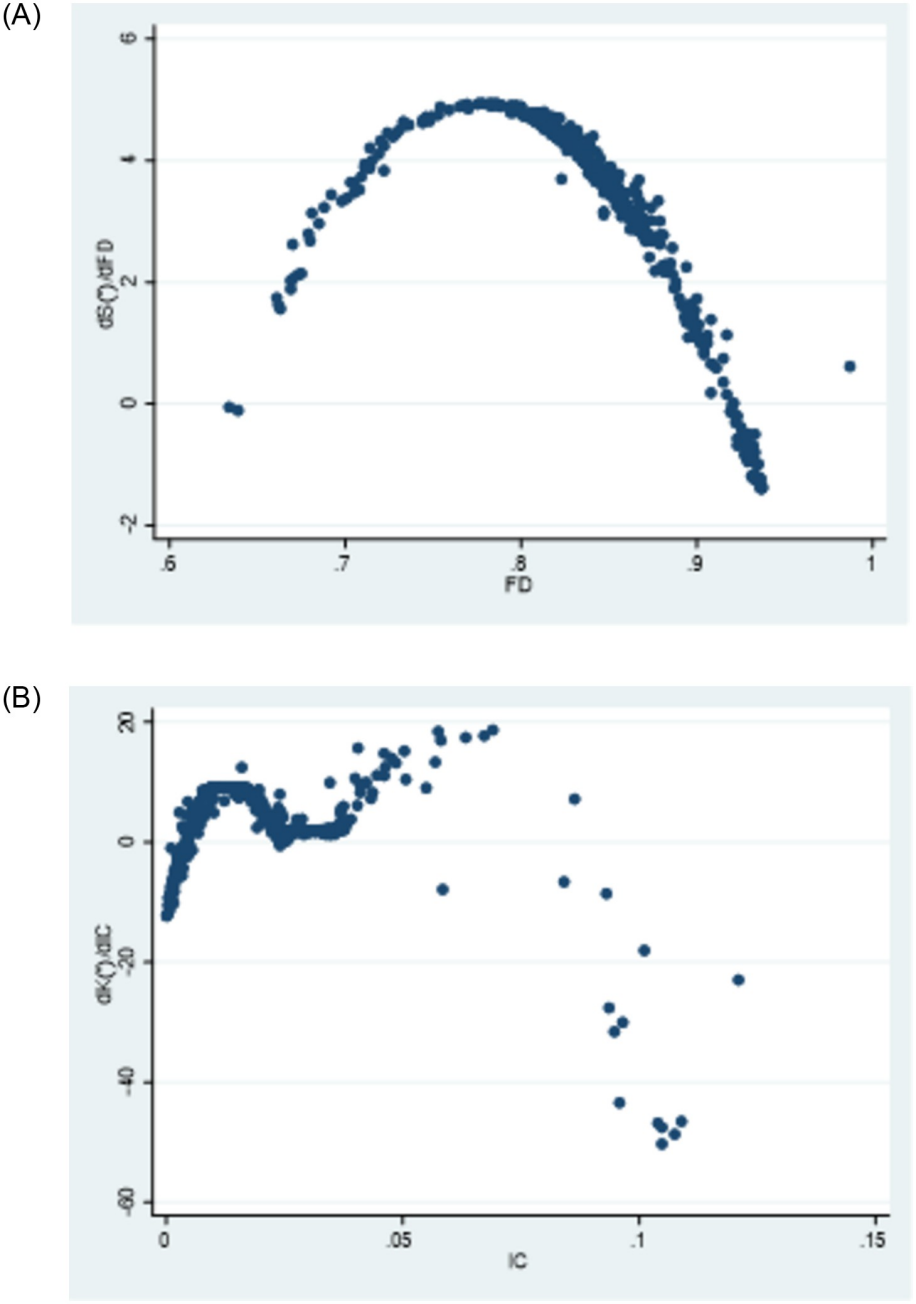


Due to the global economic downturn in 2008, FDI and international trade in many regions of China were severely affected, which also impacted the competition landscape. Local governments in China faced significant challenges in maintaining economic growth, livelihood security, and public service provision [87]. Following the approach adopted by Li et al [88], we conducted a robustness test by excluding the year 2008, which was a special year, to examine the effects of vertical competition and horizontal competition on the improvement of public service efficiency. The results of the experiment are presented in Table 9 and Fig 9. After excluding the special year, the results remain consistent with the findings from the previous analysis, indicating the robustness of the experimental results in this study.

**Table 9:** Robustness test result (2).

	(1)	(2)
	PSE	PSE
W.PSE	1.907***	-1.994***
	(0.107)	(0.106)
SEG	-467.794**	-461.004**
	(203.929)	(202.054)
ECO	0.017	0.039
	(0.208)	(0.195)
IND	0.542*	0.383
	(0.295)	(0.296)
EDU	-0.309**	-0.297**
	(0.155)	(0.143)
OPN	-1.232	-1.390
	(0.964)	(0.945)
POP	0.348	-0.262
	(1.865)	(1.743)
TEC	-0.203	-0.077
	(0.164)	(0.154)
N	510	510
R^2^	0.4728	0.4798

## 5 Conclusion and policy implications

### 5.1 Conclusion

Government and the market are two crucial mechanisms for resource allocation, and they should not only contribute to economic development but also complement each other in terms of public service provision. To investigate the impact of government and the market on public service efficiency, this study employs the theory of spatial econometrics and panel data from 30 provinces in China spanning from 2004 to 2021. A semi-parametric spatial lag model is constructed to examine the effects of market segmentation, vertical competition, and horizontal competition public service efficiency. The findings are as follows: Firstly, there is a spatial agglomeration effect in the public service efficiency of local governments in China, with notable disparities among the eastern, central, and western regions. The public service efficiency in the eastern and western regions shows significant spatial clustering, characterized by “high-high” clustering and “low-low” clustering, respectively. This discrepancy is primarily attributed to the varying levels of regional economic development, which is consistent with the findings of Wang and Li that highlight the “east high, west low” spatial imbalance in basic public service efficiency in China [89]. Overall, public service provision in China is steadily improving, and with time, the regional disparities in public service efficiency are gradually diminishing, leading to a convergence in public service efficiency across the country.

Secondly, from an overall perspective, market segmentation in China is gradually relaxing, and it has a significant negative impact on public service efficiency. However, The effects of market segmentation on public service efficiency differ significantly across regions. In the western region, market segmentation has a notable positive impact on public service efficiency because the overall economy in the western region lags behind, and local governments implement market segmentation policies that are more conducive to local economic development and improvement of public service levels. In contrast, in the developed regions of the central and eastern parts, reducing market segmentation and promoting regional integration are beneficial for local economic development. As pointed out by Xu and Fang, the degree of market segmentation between regions is influenced by the trade-offs made by local government officials to ensure successful political promotion [85, 86]. Therefore, implementing market segmentation between backward regions proves advantageous, while encouraging regional integration is more favorable in economically developed regions.

Thirdly, there is an inverted U-shaped relationship between vertical competition among governments and public service efficiency in China, with the peak value observed at 0.808. When the degree of fiscal decentralization is below 0.0808, vertical competition among governments positively impacts public service efficiency. This is because, under the fiscal decentralization system, fiscal and political incentives serve as constraints on local government behavior, leading to improved efficiency in public service provision. However, when the degree of fiscal decentralization exceeds 0.808, the partial derivative of fiscal decentralization on public service efficiency decreases or even becomes negative. The diminishing incentive effect results from the differentiation of public service provision among regions, which leads to diseconomies of scale, rent-seeking behavior, and corruption among local government officials. This suggests the presence of an optimal level of fiscal decentralization in China, which differs from the findings of Luo and Shen [44]. They concluded that the current fiscal decentralization system in China hampers the efficiency of local public service provision based on a spatial Durbin model. Conversely, Chu argues that fiscal decentralization has both positive and negative effects on the efficiency of compulsory education service provision, indicating a nonlinear relationship between fiscal decentralization and compulsory education service efficiency. Therefore, the relationship between fiscal decentralization and public service efficiency should not be simplistically characterized as consistently positive or negative, but rather as a nonlinear association [48].

Fourthly, the impact of horizontal competition among local governments in China on public service efficiency follows a wave-like oscillating curve, and its effect depends on the degree of competition between local governments and the balance of positive and negative effects on public service efficiency. The turning points are 0.011, 0.027, and 0.070, respectively. This finding supports the earlier hypothesis of this study, highlighting that government competition is dynamically influenced by internal and external factors, so its impact on public service efficiency is dynamic. Currently, the academic community acknowledges that government competition can have either a positive or negative impact on public service efficiency. The research findings presented in this study provide a fresh perspective on this issue.

In addition, the relationship between residents’ education level, science and technology, and public service efficiency shows a negative correlation. These findings echo the research of Xu and Yang, and Wang [1, 80].

### 5.2 Policy implications

To begin, it is crucial to acknowledge the regional heterogeneity in public service efficiency and implement tailored public service policies accordingly. In the eastern region, characterized by rapid economic development and consistently high levels of public service provision, a greater emphasis should be placed on evaluating the quantity and quality of public services. Encouraging competition among local governments can serve as a catalyst for meeting the diverse demands of the public more effectively. In the central region, where significant disparities exist among provinces and the overall level of public services is lower compared to the eastern region, targeted measures are necessary. The central government should augment transfer payments to the western region, providing increased financial support to ensure the provision of basic public services. This support should be focused on essential areas like compulsory education and healthcare.

Then, strengthen cross-regional cooperation and enhance the synergistic effect between government competition and public service efficiency. The following measures can be implemented: (1) Promoting regional integration. Given the long-standing issue of market segmentation in China, it is crucial for the central government to guide and actively promote regional integration strategies at the policy level. This entails reducing the adverse impact of geographical boundaries on the market. By strengthening cooperation between provinces and leveraging complementary advantages, regional integration can effectively address market segmentation and facilitate the smooth flow of goods, services, and resources across regions. (2) Incorporating social governance and healthcare in performance evaluation. To enhance the synergistic effect between government competition and public service efficiency, it is essential to include social governance, healthcare, and other aspects in the performance evaluation system for local officials. This ensures that local governments strike a balance between improving public services and pursuing GDP growth. By incorporating these factors into performance evaluations, officials will be motivated to prioritize public service improvement alongside economic development. (3) Optimizing fiscal expenditure structure. Analysis indicates that the disproportionate allocation of fiscal resources to economic development by local governments hampers the improvement of public service efficiency. To address this, local governments should optimize the structure of fiscal expenditures. This includes establishing comprehensive assessment indicators for public service satisfaction and guiding local governments to allocate more fiscal resources to public service provision. (4) Providing political incentives and establish benchmark effects: To maximize the spatial interaction effect and incentivize cities to improve public service provision, it is crucial to provide more political incentives to cities that excel in delivering public services. Establishing benchmark effects can motivate surrounding cities to enhance the quality of their own public service provision, thus driving overall improvements. This approach fosters healthy competition and promotes the highest quality of public service provision [90].

Finally, it is important to have a comprehensive understanding of the path differences in fiscal decentralization and the impact of intergovernmental competition on public service efficiency. The nonlinear impact of fiscal decentralization and intergovernmental competition on public service efficiency not only confirms the validity of “central planning and local implementation” for public service provision within China’s decentralized system, but also emphasizes the need to delegate more responsibilities for public service provision to local governments in the upcoming round of fiscal and tax system reforms. However, alongside the delegation of public service provision responsibilities, it is vital to reform the government’s performance evaluation system and enhance accountability for public service provision by local governments. This serves as the fundamental solution to improving government administrative capacity and ensuring effective public service delivery. Moreover, it is worth considering that there may exist an optimal level of fiscal decentralization in China. Hence, the central and local governments should actively seek the optimal balance point in practice. This involves leveraging the incentive effects of decentralization reforms on local governments while simultaneously implementing regulations to ensure fair and healthy competition among local entities. By striking the right balance, policymakers can effectively reshape the fiscal relationship between the central and local governments during the new round of fiscal and tax system reforms.

### 5.3 Shortcomings and prospects of research

Currently, spatial econometric models primarily rely on two types of data: cross-sectional data and panel data. The semi-parametric model of spatial lag is commonly used with cross-sectional data, but it has limitations in terms of capturing temporal dynamics. Although many scholars have attempted to address temporal fluctuations by calculating average values of variables across multiple time periods in cross-sectional data, this approach often results in a loss of valuable behavioral information with temporal evolution characteristics, making it challenging to accurately uncover the true relationships between variables with spatiotemporal characteristics. On the other hand, some researchers have employed panel data to tackle the issues associated with temporal changes. However, they have not yet fully resolved the nonlinearity problem that may exist between variables in this context. Consequently, further research is warranted to develop spatial panel data models that incorporate semi-parametric designs, thus allowing for a more comprehensive analysis of spatiotemporal relationships [91].

Additionally, this study confirms the inverted U-shaped impact of fiscal decentralization on public service efficiency, indicating the existence of an optimal level of fiscal decentralization in China. However, further analysis and demonstration of this conclusion have not been conducted. Subsequent research can employ threshold models to further investigate the potential temporal changes and threshold effects between fiscal decentralization and public service efficiency.

## Supporting information

S1 File Original data. (DOCX)

S1 Data (DTA)

S1 Dataset (ZIP)
