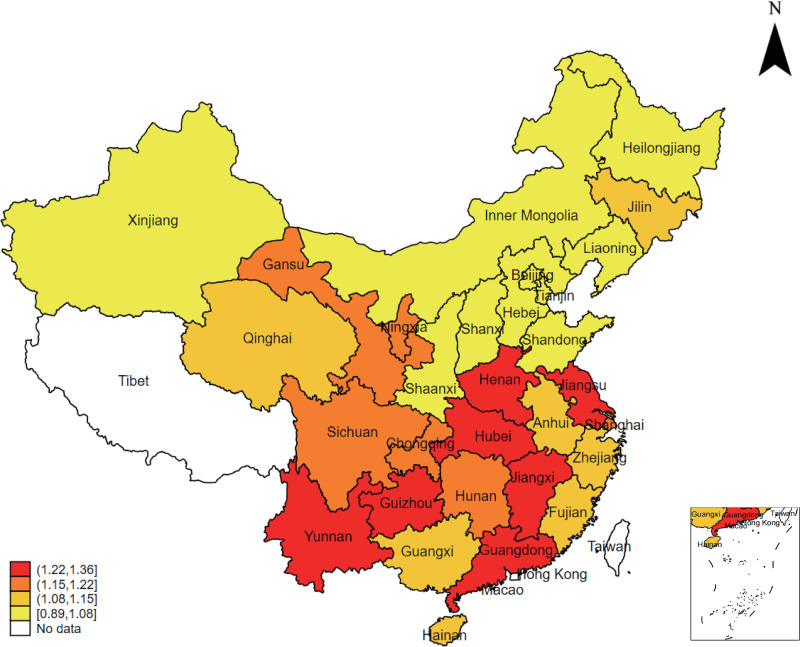# Correction: A study of market segmentation, government competition, and public service efficiency in China: Based on a semi-parametric spatial lag model

**DOI:** 10.1371/journal.pone.0320963

**Published:** 2025-03-25

**Authors:** Lan Yao, Ruoyu Luo, Xiaoqin Yi

In Fig 1, the image of the South China Sea and its Nine-dashed Line at the lower right is omitted. Please see the correct Fig 1 here,

**Fig 1:**