# Supplementary material for: A study of market segmentation, government competition, and public service efficiency in China: Based on a semi-parametric spatial lag model
Source: PLoS One. 2024 Apr 16;19(4):e0297446. doi: 10.1371/journal.pone.0297446 (PMC11020847; doi:10.1371/journal.pone.0297446)
Supplement: S1 File — (DOCX) [file pone.0297446.s001.docx]

Financial Disclosure

"Humanities and Social Science Fund of the Ministry of Education of China, Grant No. 19YJA630051, Professor Ruoyu Luo."
